# Folsom's Improved Atmospheric Dental Plates

**Published:** 1872-03

**Authors:** 


### ARTICLE VII.

### *Folsom's Improved Atmospheric Dental Plates.*

In reply to our correspondents, to whom our name has been given as reference, and who desire to learn that we know about Dr. Folsom's improvement, we publish the following as the sum total of our personal knowledge in the matter:

On the 1st day of January, 1867, letters patent were granted to N. T. Folsom, for certain improvements in atmospheric dental plates. These improvements consist in what Dr. F. terms packing ridges, either hard or soft, extending round the border of the plate.

Dr. Straight also received a patent, November, 1869, for the attachment of a soft elastic edge on the rear of a rubber plate.

A patent was also granted Dr. Hurd, September, 1866, for the mode of constructing a flange, either hard or soft, around the edge, or border of a rubber plate.

Patent was also granted Dr. Twitchell, for a soft rubber cushion, or flexible lining for dental plates.

The end sought to be attained by these various improvements being the same, viz., to secure a plate firmly in the mouth, so that it will not drop while eating or speaking; we mention them, in this connection, none of these so-called improvements seem to have met with much favor with the profession so far as we have been able to learn. Dr. Folsom's is undoubtedly of the most practical value, and hence has been more generally adopted. The hard ridge on rubber plates, described by Dr. Folsom some three years since, and published in the first number of this *Journal*, has been used by the profession here, for five or six years past, and by many thought to be of much practical value.

The soft ridge was brought before the dentists of our city in March last, by Dr. Folsom himself. He put his soft ridge on a set of plates in our office. The patient, an old gentleman for whom several unsuccessful attempts had been made to supply with artificial teeth, with which he could eat. For the first few days after the soft edge was put on the plates, the patient seemed to get along quite well, but in less than two weeks we met him, and learned to our surprise and regret that he could not do any better, if as well, with his teeth as he could before any improvement was made on them. He gave them up and has not worn them since. In justice to Dr. F., we should here state what we believe to be true, that the failure in this patient's case was due to causes over which soft ridges, flanges, soft edges, or anything short of a rivet through the plate could not prevail.

We have never applied Dr. F's improvement; have never seen it applied; have heard of but two cases, since his visit here, where it has been used, and in both of these cases it was a failure; it may have been the fault of the operator. We have no doubt, if properly applied to a plate in extreme cases, it may serve a good purpose, by aiding the retention of the plates in position till the patient has become accustomed to them, and learned the art of using them. We do not think it can serve more than a temporary purpose. From personal observation we find that soft rubber soon deteriorates when worn in the mouth.

The following directions for constructing a plate with the hard ridge are taken from Dr. Folsom's circular: E.

"To prepare a model for an upper set, with my improvement, take an impression in wax; trim off the surplus; press out the wax that comes against the labial surface of the dental ridge, to give room for plaster; then cool the wax; you now have a cup suited to the case, in which take the impression in plaster, mixed in a solution of sulphate of potassa (Dr. Chamberlain's rule is one-half an once to a quart of water—sometimes more and sometimes less, according to the plaster); varnish with ethereal varnish. Mix the plaster for the model with the same solution; dip the impression in water, and immediately pour the plaster. The next step is to examine the mouth, to ascertain where the edge of the plate will extend, then mark the line on the model; examine the mouth again to ascertain the yielding nature of the parts of this same line, and note the hard and soft places; then with a suitable instrument, cut a groove in the plaster model, along the entire line of the edge of the plate, one-twentieth of an inch wide, varying in depth from one-sixteenth to one sixtieth of an inch. As a general rule, I commence cutting the groove in the rear of the tuberosity on the right side of the mouth; at this point deep, then shallow till I reach the soft part at the side of the back of the mouth, which I cut deeper than any other point, then shallow till I reach the corresponding point on the opposite side; from the rear of the tuberosities to the canine teeth, comparatively shallow; around in front, from canine to canine, quite deep. I endeavor to cut a well-defined groove-

A plate made on this cast, if not injured in vulcanizing or otherwise, will fit not only the mouth, so that atmos. pheric pressure is obtained, but has a packing ridge encircling it which prevents ingress of air under it, and thereby secures the pressure on the outside. I make the ridge as high, all round the plate, as possible, and not pinch the flesh.

To make a plate with the soft or elastic Packing Ridge; the model is prepared as for the hard ridge. Cut the pat tern plate short, so that it will not reach the groove by at the least one-eighth of an inch. Wax up the job taking care care not to extend the wax beyond the pattern. Take a strip of soft rubber, wide enough to just cover the groove and reach the waxing, lay it along the edge of the pattern plate in its place, and join it to the waxing with melted wax. Now mix some plaster, and cover the soft rubber, and let the plaster extend down the sides of the cast or model. It is now ready to flask. After opening the flasks, wash out with boiling water all the wax from the soft rubber; take a strip of soft rubber, and pack on the soft rubber that is confined in the plaster; then take a strip of vulcanite, wide enough to cover the soft rubber, and pack it on the soft all around. Now pack and vulcanize as usual.

In finishing a plate with either hard or soft ridges, be careful and not mar the packing. Trim the soft rubber with sharp scissors.

In fitting a plate in the mouth with the hard packing, reduce the ridge where it is too high, by scraping with a sharp knife cautiously. It is generally well to reduce the plate inside of the ridge with a hoe-shaped instrument, at the time the ridge is reduced.

The same directions apply to the under plates, excepting the ridge is not more that half as high."—*Missouri Dental Journal.*

